# Bis{μ-1,3-bis­[dimeth­yl(pyridin-3-yl)sil­yl]propane-κ^2^*N*:*N*′}bis­[di­iodido­zinc(II)] from synchrotron data

**DOI:** 10.1107/S2414314626001835

**Published:** 2026-02-24

**Authors:** Jiyeong Song, Dongwon Kim, Young-A Lee

**Affiliations:** a Department of Chemistry, Jeonbuk National University, Jeonju 54896, Republic of Korea; b Beamline Department, Pohang Acceleratory Laboratory, Pohang 37673, Republic of Korea; Purdue University, USA

**Keywords:** crystal structure, metallamacrocyclic complex, 1,3-bis­(di­methyl­silyl-*3*-pyridine)­propane ligand, zinc(II) complexes, Synchrotron data

## Abstract

The structure of the title compound, [Zn_2_I_4_(C_34_H_52_N_4_Si_4_)_2_], has been determined from synchrotron data, λ = 0.70000 Å. The complete metallacyclic mol­ecule is generated by crystallographic inversion symmetry, with the Zn^II^ ion located in a general position. The 1,3-bis­(di­methyl­silyl-3-pyridine)­propane ligand binds to two zinc(II) ions in a horse-shoe fashion, resulting in formation of a dimeric 24-membered macrocycle. The Zn^II^ ion has a typical tetra­hedral geometry *via* two iodide ions and two N donor atoms of the 1,3-bis­(di­methyl­silyl-3-pyridine)­propane ligand. The macrocyclic dimers inter­act *via* weak inter­actions [I⋯H (H_3_CSi-) = 3.08, 3.27 Å].

## Abstract 2

In the title Zn^II^ complex, the 1,3-bis­(di­methyl­silyl-3-pyridine)­propane ligands bind to two Zn^II^ ions in a horse-shoe fashion to form a centrosymmetric dimeric 24-membered macrocycle, which further assembles into a supra­molecular structure *via* weak I⋯H inter­actions.

## Graphical abstract

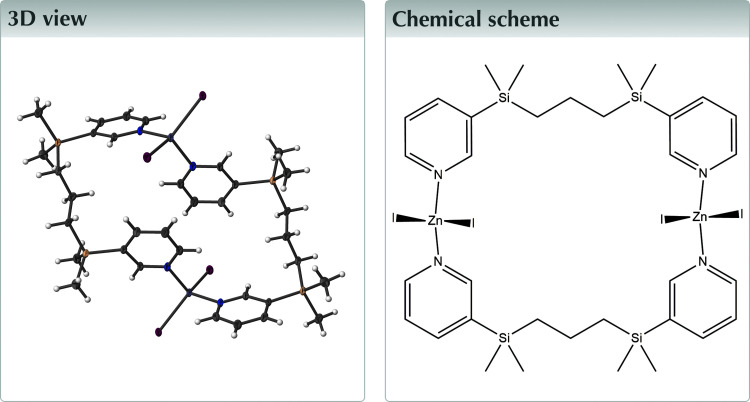

## Structure description

Designed horse-shoe bidentate N-donors provide, *via* the introduction of appropriate metal cations, wider opportunities for task-specific metallacycles as receptors (Na *et al.*, 2008 ▸). Specifically, Zn^II^ complexes of functional N-donor ligands have been extensively examined for metallo-enzymes, zinc finger proteins, transmetallation, recognition, photoluminescence (PL), and catalysts (Porchia *et al.*, 2020 ▸). In particular, arrays of macrocyclic mol­ecular units, especially after the emergence of additional functionalities, have attracted crystal engineers for the past decade (Lindoy *et al.*, 2013 ▸) in the fields of mol­ecular adsorption, recognition, ion exchange, confinement catalysis, and luminescent chemosensing. Here, we report the crystal structure of a Zn^II^ 24-membered macrocycle, [ZnI_2_(*L*)]_2_, *via* self-assembly of ZnI_2_ with 1,3-bis­(di­methyl­silyl-*m*-pyridine)­propane (*L*) as a hemi-circular bidentate ligand. The incorporation of the flexible di­methyl­silyl spacers in *L* plays a crucial role in the assembly process. This moiety provides the necessary conformational freedom and specific curvature to accommodate the tetra­hedral coordination geometry of the Zn^II^ ions, thereby facilitating the formation of a discrete, strain-free macrocyclic architecture without significant steric hindrance.

**Figure 1:**
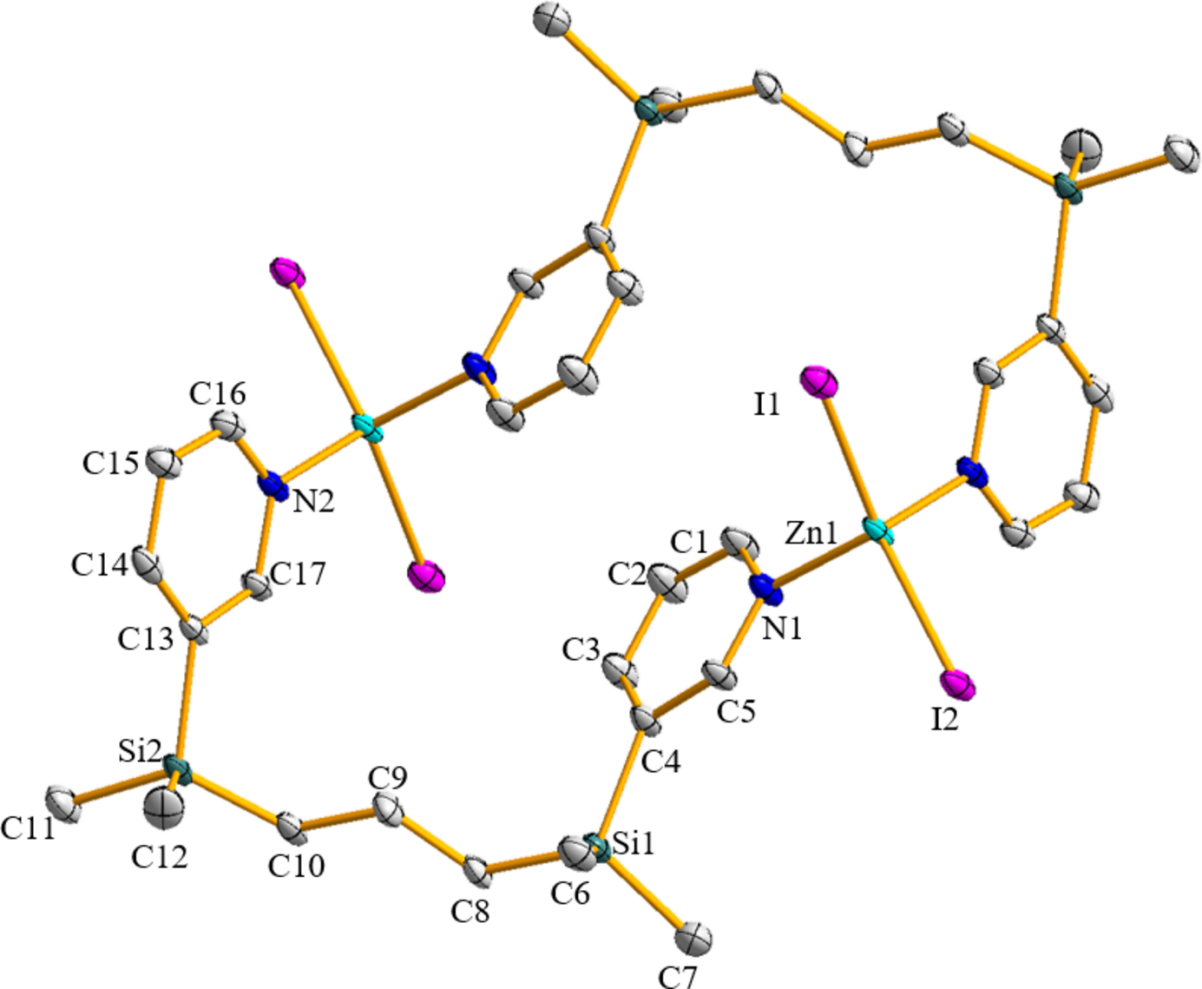
A view of the mol­ecular structure of the title compound, showing the macrocyclic dimers with displacement ellipsoids drawn at the 30% probability level. For clarity, H atoms have been omitted. Symmetry operation used to generate equivalent atoms: 1 − *x*, 1 − *y*, 1 − *z*.

The defining structural feature of the title complex is the formation of a 24-membered centrosymmetric macro­cyclo­dimer. The relevant bond lengths and angles are listed in Table 1 ▸. The local geometry around the Zn^II^ cation approximates to a typical tetra­hedral arrangement with two *N* donors from two ligands [N—Zn—N = 101.75 (11)°] and two iodide ions [I—Zn—I = 121.03 (2)°]. For the 24-membered macrocycle, the intra­molecular Zn⋯Zn separation distance is 8.118 (2) Å, and the shortest distance [C1⋯C2^iii^ or C2⋯C1^iii^; symmetry code: (iii) 1 − *x*, 1 − *y*, 1 − *z*] between two pyridyl moieties is 3.404 (7) Å. Fig. 1 ▸ illustrates the mol­ecular structure of the centrosymmetric dimer. The propyl linkers adopt an extended all-anti conformation with close to 180° torsion angles, effectively minimizing intra­molecular steric strain within the macrocycle. The flexible silicon bridges accommodate a slightly distorted tetra­hedral geometry around the Zn^II^ center, allowing the formation of a discrete, strain-free assembly. This arrangement is further stabilized by the specific inter­molecular inter­actions described below.

**Figure 2:**
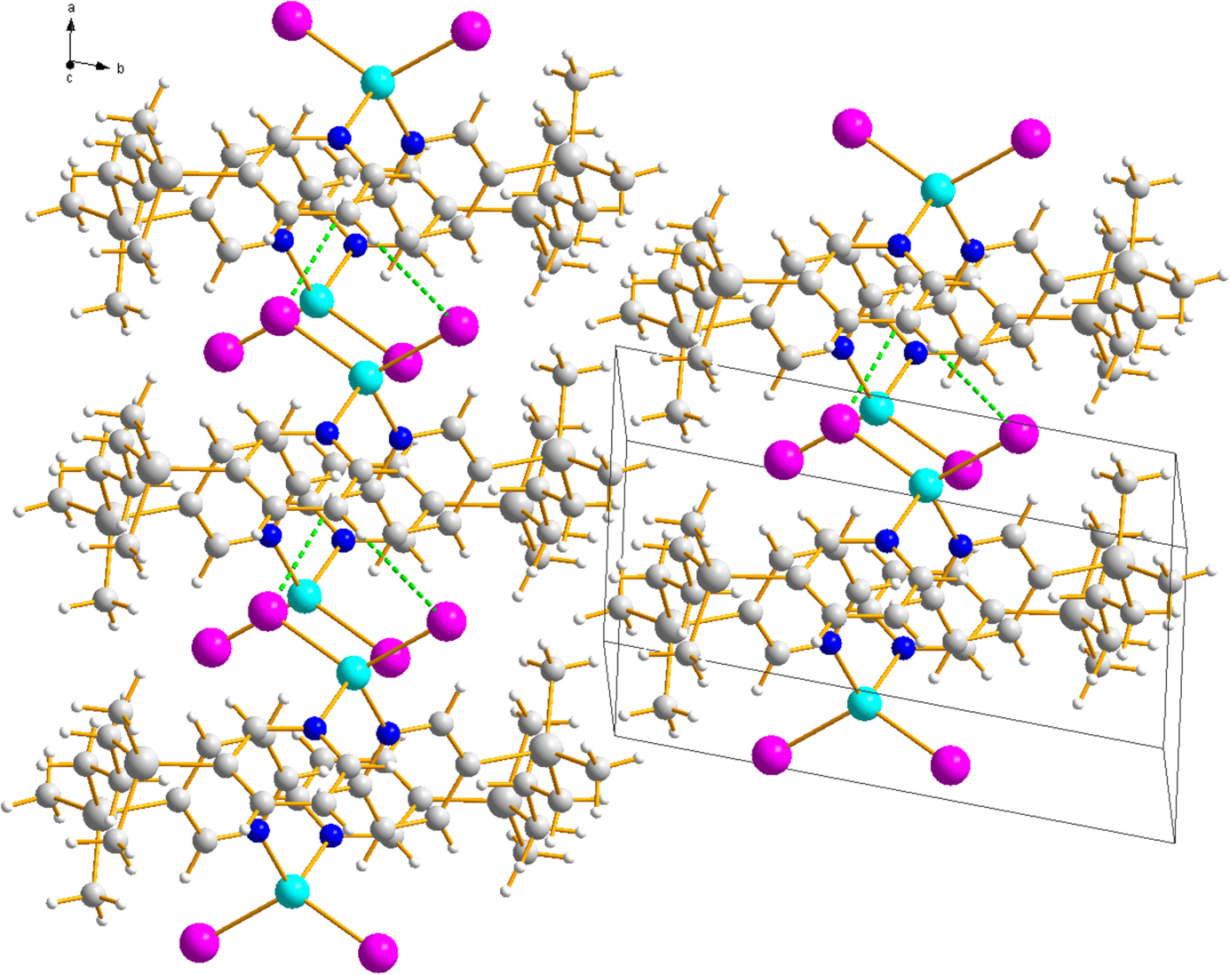
The crystal packing in title compound. Dashed lines represent C—H⋯I inter­actions.

The crystal packing of the title complex is primarily consolidated by a network of weak inter­molecular C—H⋯I hydrogen bonds involving the pyridyl ligands (Table 2 ▸ and Fig. 2 ▸). Specifically, the pyridyl ring hydrogen atom H1 forms a hydrogen bond with the iodide atom I1 of an adjacent mol­ecule. In addition, the other pyridyl hydrogen atom H15 also participates in a significant inter­action with the iodide ligand. Although classical π–π stacking inter­actions are not prominent, these multiple weak C—H⋯I inter­actions serve as the principal forces that connect the layers and enhance the overall stability of the mol­ecular arrangement in the solid state.

A search of the Cambridge Structural Database (CSD, version 6.00 with updates through April 2025; Groom *et al.*, 2016 ▸) indicated that Hg^II^ complexes with the 1,3-bis­(di­methyl­silyl-3-pyridine)­propane ligand had been reported previously. These complexes have been studied for straightforward formation of dianionic acetonylates (Hong *et al.*, 2021 ▸). Furthermore, including the work by Na *et al.* (2008 ▸), a total of 14 complexes involving a cognate ligand, 1,3-bis­[dimeth­yl(pyridin-3-yl)sil­yl]ethane, have been reported in the CSD. However, no corresponding Zn^II^ complex with the ligand has been reported and the title compound was newly synthesized for this research.

## Synthesis and crystallization

The title Zn^II^ complex was prepared as follows. A solution was prepared by dissolving ZnI_2_ (0.02 mmol) in ethanol, and another by dissolving 1,3-bis­(di­methyl­silyl-*3*-pyridine)­propane (0.02 mmol) in ethanol. Slow diffusion of the two solutions over several days afforded colorless needle-shaped crystals suitable for X-ray diffraction. Yield: 91.4%. FT–IR (KBr pellet, cm^−1^): 3436 (*s*), 2903 (*m*), 1589 (*m*), 1399 (*m*), 1255 (*m*), 1133 (*m*), 907 (*m*), 842 (*m*), 818 (*m*), 801 (*m*), 703 (*m*). ^1^H NMR (400 MHz, Me_2_SO-*d*_6_, ppm): 8.59 (*d*, *J* = 1.4 Hz, 2H), 8.54 (*dd*, *J* = 4.9, 1.9 Hz, 2H), 7.82 (*dt*, *J* = 7.5, 1.9 Hz, 2H), 7.35 (*dd*, *J* = 7.5, 4.9 Hz, 2H), 1.50 −1.20 (*m*, 2H), 0.81 (*dd*, *J* = 6.9, 3.8 Hz, 4H), 0.23 (*s*, 12H). Analysis calculated for Zn_2_Si_4_N_4_I_4_C_34_H_52_·2.5H_2_O (reflecting hygroscopic moisture) (*M* = 1312.57): C = 31.11%; H = 4.38%; N = 4.27%. Found: C = 31.10%; H = 4.11%; N = 4.38%.

## Refinement

Crystal data, data collection and structure refinement details are summarized in Table 3 ▸.

## Supplementary Material

Crystal structure: contains datablock(s) I. DOI: 10.1107/S2414314626001835/zl4091sup1.cif

Structure factors: contains datablock(s) I. DOI: 10.1107/S2414314626001835/zl4091Isup2.hkl

CCDC reference: 2531921

Additional supporting information:  crystallographic information; 3D view; checkCIF report

## Figures and Tables

**Table 1:** Selected geometric parameters (Å, °)

Zn1—I1	2.5680 (7)	Zn1—N1	2.051 (3)
Zn1—I2	2.5688 (9)	Zn1—N2^i^	2.058 (3)
			
N1—Zn1—N2^i^	101.75 (11)	N1—Zn1—I2	103.93 (8)
N1—Zn1—I1	112.18 (9)	N2^i^—Zn1—I2	110.36 (8)
N2^i^—Zn1—I1	105.99 (8)	I1—Zn1—I2	121.03 (2)

Symmetry code: (i) 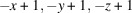.

**Table 2:** Hydrogen-bond geometry (Å, °)

*D*—H⋯*A*	*D*—H	H⋯*A*	*D*⋯*A*	*D*—H⋯*A*
C1—H1⋯I1^ii^	0.95	3.08	3.805 (4)	135
C15—H15⋯I2^iii^	0.95	3.27	3.756 (3)	114

Symmetry codes: (ii) 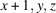; (iii) 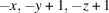.

**Table 3:** Experimental details

Crystal data	
Chemical formula	[Zn_2_I_4_(C_34_H_52_N_4_Si_4_)_2_]
*M*_r_	1267.49
Crystal system, space group	Triclinic, *P* 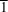
Temperature (K)	100
*a*, *b*, *c* (Å)	6.6360 (13), 13.531 (3), 14.416 (3)
α, β, γ (°)	107.03 (3), 91.52 (3), 100.29 (3)
*V* (Å^3^)	1213.5 (5)
*Z*	1
Radiation type	Synchrotron, λ = 0.700 Å
μ (mm^−1^)	3.48
Crystal size (mm)	0.15 × 0.11 × 0.07

Data collection	
Diffractometer	Rayonix MX225HS CCD area detector
Absorption correction	Empirical (using intensity measurements) (*HKL3000sm *SCALEPACK**; Otwinowski *et al.*, 2003 ▸)
*T*_min_, *T*_max_	0.942, 1.000
No. of measured, independent and observed [*I* > 2σ(*I*)] reflections	13971, 7000, 6774
*R*_int_	0.020
(sin θ/λ)_max_ (Å^−1^)	0.704

Refinement	
*R*[*F*^2^ > 2σ(*F*^2^)], *wR*(*F*^2^), *S*	0.042, 0.126, 1.15
No. of reflections	7000
No. of parameters	222
H-atom treatment	H-atom parameters constrained
Δρ_max_, Δρ_min_ (e Å^−3^)	1.75, −1.60

Computer programs: *PAL BL2D-SMDC Program* (Shin *et al.*, 2025 ▸), *HKL3000sm* (Otwinowski *et al.*, 2003 ▸), *SHELXT2018* (Sheldrick, 2015*a* ▸), *SHELXL2025* (Sheldrick, 2015*b* ▸), *DIAMOND 4* (Putz & Brandenburg, 2014 ▸) and *publCIF* (Westrip, 2010 ▸).

## full crystallographic data

### Bis{µ-1,3-bis[dimethyl(pyridin-3-yl)silyl]propane-κ2N:N'}bis[diiodidozinc(II)] Crystal data

**Table:**

[Zn_2_I_4_(C_34_H_52_N_4_Si_4_)_2_]	*Z* = 1
*M**_r_* = 1267.49	*F*(000) = 612
Triclinic, *P*1	*D*_x_ = 1.734 Mg m^−^^3^
*a* = 6.6360 (13) Å	Synchrotron radiation, λ = 0.700 Å
*b* = 13.531 (3) Å	Cell parameters from 25750 reflections
*c* = 14.416 (3) Å	θ = 0.4–29.5°
α = 107.03 (3)°	µ = 3.48 mm^−^^1^
β = 91.52 (3)°	*T* = 100 K
γ = 100.29 (3)°	Block, colorless
*V* = 1213.5 (5) Å^3^	0.15 × 0.11 × 0.07 mm

### Bis{µ-1,3-bis[dimethyl(pyridin-3-yl)silyl]propane-κ2N:N'}bis[diiodidozinc(II)] Data collection

**Table:**

Rayonix MX225HS CCD area detector diffractometer	6774 reflections with *I* > 2σ(*I*)
Radiation source: PLSII 2D bending magnet	*R*_int_ = 0.020
ω scan	θ_max_ = 29.6°, θ_min_ = 1.5°
Absorption correction: empirical (using intensity measurements) (*HKL3000sm Scalepack*; Otwinowski *et al.*, 2003)	*h* = −9→9
*T*_min_ = 0.942, *T*_max_ = 1.000	*k* = −19→19
13971 measured reflections	*l* = −20→20
7000 independent reflections	

### Bis{µ-1,3-bis[dimethyl(pyridin-3-yl)silyl]propane-κ2N:N'}bis[diiodidozinc(II)] Refinement

**Table:**

Refinement on *F*^2^	Secondary atom site location: difference Fourier map
Least-squares matrix: full	Hydrogen site location: inferred from neighbouring sites
*R*[*F*^2^ > 2σ(*F*^2^)] = 0.042	H-atom parameters constrained
*wR*(*F*^2^) = 0.126	*w* = 1/[σ^2^(*F*_o_^2^) + (0.0818*P*)^2^ + 1.9207*P*] where *P* = (*F*_o_^2^ + 2*F*_c_^2^)/3
*S* = 1.15	(Δ/σ)_max_ = 0.001
7000 reflections	Δρ_max_ = 1.75 e Å^−^^3^
222 parameters	Δρ_min_ = −1.60 e Å^−^^3^
0 restraints	Extinction correction: SHELXL2025/1 (Sheldrick 2015b), Fc^*^=kFc[1+0.001xFc^2^λ^3^/sin(2θ)]^-1/4^
Primary atom site location: dual	Extinction coefficient: 0.0393 (15)

### Bis{µ-1,3-bis[dimethyl(pyridin-3-yl)silyl]propane-κ2N:N'}bis[diiodidozinc(II)] Special details

**Table:**

Geometry. All esds (except the esd in the dihedral angle between two l.s. planes) are estimated using the full covariance matrix. The cell esds are taken into account individually in the estimation of esds in distances, angles and torsion angles; correlations between esds in cell parameters are only used when they are defined by crystal symmetry. An approximate (isotropic) treatment of cell esds is used for estimating esds involving l.s. planes.

### Bis{µ-1,3-bis[dimethyl(pyridin-3-yl)silyl]propane-κ2N:N'}bis[diiodidozinc(II)] Fractional atomic coordinates and isotropic or equivalent isotropic displacement parameters (Å^2^)

**Table:**

	*x*	*y*	*z*	*U*_iso_*/*U*_eq_	
I1	0.02739 (3)	0.60462 (2)	0.71151 (2)	0.02188 (9)	
I2	−0.04076 (3)	0.28606 (2)	0.75347 (2)	0.02388 (9)	
Zn1	0.18295 (5)	0.44768 (3)	0.72356 (2)	0.01825 (11)	
Si1	0.20755 (13)	0.11080 (7)	0.36959 (6)	0.01815 (17)	
Si2	0.29081 (13)	0.19038 (7)	0.00126 (6)	0.01876 (17)	
N1	0.3265 (4)	0.3852 (2)	0.6019 (2)	0.0211 (5)	
N2	0.5728 (4)	0.4903 (2)	0.17215 (19)	0.0188 (5)	
C1	0.5162 (5)	0.4319 (3)	0.5905 (2)	0.0251 (6)	
H1	0.579527	0.495898	0.638031	0.030*	
C9	0.2785 (5)	0.1499 (3)	0.1859 (2)	0.0221 (6)	
H9A	0.319184	0.225709	0.222588	0.027*	
H9B	0.127944	0.134290	0.169839	0.027*	
C10	0.3857 (5)	0.1284 (2)	0.0903 (2)	0.0206 (6)	
H10A	0.364468	0.051314	0.059107	0.025*	
H10B	0.535313	0.155339	0.105999	0.025*	
C11	0.4132 (6)	0.1526 (3)	−0.1155 (3)	0.0291 (7)	
H11A	0.348469	0.178332	−0.163340	0.044*	
H11B	0.395268	0.075655	−0.140057	0.044*	
H11C	0.560138	0.183835	−0.104471	0.044*	
C12	0.0055 (5)	0.1505 (3)	−0.0225 (3)	0.0300 (7)	
H12A	−0.041008	0.178965	−0.072628	0.045*	
H12B	−0.058849	0.178144	0.037643	0.045*	
H12C	−0.033317	0.073428	−0.044895	0.045*	
C13	0.3555 (4)	0.3384 (2)	0.0570 (2)	0.0177 (5)	
C14	0.2430 (5)	0.4065 (3)	0.0321 (2)	0.0214 (6)	
H14	0.130060	0.378651	−0.016029	0.026*	
C15	0.2954 (5)	0.5143 (3)	0.0771 (2)	0.0233 (6)	
H15	0.219466	0.560577	0.060122	0.028*	
C16	0.4601 (5)	0.5533 (2)	0.1470 (2)	0.0210 (6)	
H16	0.494752	0.627136	0.178439	0.025*	
C17	0.5198 (5)	0.3852 (2)	0.1279 (2)	0.0193 (5)	
H17	0.598922	0.340795	0.146053	0.023*	
C2	0.6227 (5)	0.3902 (3)	0.5118 (3)	0.0284 (7)	
H2	0.757203	0.424652	0.505495	0.034*	
C3	0.5296 (5)	0.2971 (3)	0.4423 (2)	0.0239 (6)	
H3	0.600523	0.267856	0.387566	0.029*	
C4	0.3325 (5)	0.2458 (2)	0.4519 (2)	0.0192 (5)	
C5	0.2384 (5)	0.2944 (2)	0.5337 (2)	0.0196 (6)	
H5	0.103813	0.261679	0.541876	0.024*	
C6	−0.0741 (5)	0.1046 (3)	0.3501 (3)	0.0253 (6)	
H6A	−0.139978	0.033687	0.309674	0.038*	
H6B	−0.096958	0.156130	0.317310	0.038*	
H6C	−0.133232	0.120667	0.413133	0.038*	
C7	0.2577 (6)	0.0153 (3)	0.4333 (3)	0.0296 (7)	
H7A	0.177913	−0.054853	0.398498	0.044*	
H7B	0.217186	0.037456	0.499969	0.044*	
H7C	0.404478	0.012956	0.434917	0.044*	
C8	0.3300 (5)	0.0847 (2)	0.2514 (2)	0.0202 (5)	
H8A	0.480997	0.098998	0.265646	0.024*	
H8B	0.286597	0.008978	0.214817	0.024*	

### Bis{µ-1,3-bis[dimethyl(pyridin-3-yl)silyl]propane-κ2N:N'}bis[diiodidozinc(II)] Atomic displacement parameters (Å^2^)

**Table:**

	*U*^11^	*U*^22^	*U*^33^	*U*^12^	*U*^13^	*U*^23^
I1	0.01598 (12)	0.02058 (13)	0.02297 (13)	−0.00243 (8)	0.00322 (8)	0.00066 (8)
I2	0.02429 (13)	0.01873 (13)	0.01943 (13)	−0.00750 (8)	0.00749 (8)	−0.00180 (8)
Zn1	0.01568 (17)	0.01609 (17)	0.01450 (17)	−0.00495 (12)	0.00393 (12)	−0.00377 (12)
Si1	0.0185 (4)	0.0162 (4)	0.0140 (3)	−0.0020 (3)	0.0017 (3)	−0.0012 (3)
Si2	0.0167 (4)	0.0176 (4)	0.0140 (3)	−0.0055 (3)	0.0015 (3)	−0.0023 (3)
N1	0.0173 (11)	0.0201 (12)	0.0165 (11)	−0.0059 (9)	0.0047 (9)	−0.0032 (9)
N2	0.0165 (11)	0.0169 (11)	0.0152 (10)	−0.0042 (9)	0.0034 (8)	−0.0030 (9)
C1	0.0202 (14)	0.0231 (14)	0.0214 (14)	−0.0087 (12)	0.0063 (11)	−0.0025 (12)
C9	0.0244 (14)	0.0237 (14)	0.0159 (12)	0.0058 (12)	0.0057 (11)	0.0013 (11)
C10	0.0185 (13)	0.0191 (13)	0.0161 (12)	−0.0039 (10)	0.0029 (10)	−0.0027 (10)
C11	0.0325 (17)	0.0271 (16)	0.0197 (14)	0.0001 (14)	0.0069 (13)	−0.0020 (12)
C12	0.0187 (14)	0.0339 (18)	0.0303 (17)	−0.0111 (13)	−0.0030 (12)	0.0089 (15)
C13	0.0156 (12)	0.0173 (12)	0.0133 (11)	−0.0034 (10)	0.0042 (9)	−0.0025 (9)
C14	0.0155 (12)	0.0250 (14)	0.0161 (12)	−0.0026 (11)	0.0026 (10)	−0.0017 (11)
C15	0.0223 (14)	0.0208 (14)	0.0220 (14)	0.0012 (11)	0.0055 (11)	0.0006 (11)
C16	0.0206 (13)	0.0166 (13)	0.0208 (13)	−0.0008 (11)	0.0051 (11)	0.0004 (10)
C17	0.0194 (13)	0.0162 (12)	0.0166 (12)	−0.0030 (10)	0.0024 (10)	0.0000 (10)
C2	0.0204 (14)	0.0298 (17)	0.0235 (15)	−0.0070 (13)	0.0076 (12)	−0.0028 (13)
C3	0.0183 (13)	0.0245 (15)	0.0203 (14)	−0.0038 (12)	0.0071 (11)	−0.0024 (12)
C4	0.0169 (12)	0.0191 (13)	0.0152 (12)	−0.0036 (10)	0.0020 (10)	−0.0006 (10)
C5	0.0158 (12)	0.0169 (13)	0.0184 (13)	−0.0053 (10)	0.0043 (10)	−0.0019 (10)
C6	0.0204 (14)	0.0238 (15)	0.0252 (15)	−0.0029 (12)	0.0025 (11)	0.0016 (12)
C7	0.0381 (19)	0.0221 (15)	0.0244 (15)	0.0004 (14)	−0.0008 (13)	0.0044 (12)
C8	0.0214 (13)	0.0211 (13)	0.0127 (12)	0.0014 (11)	0.0029 (10)	−0.0017 (10)

### Bis{µ-1,3-bis[dimethyl(pyridin-3-yl)silyl]propane-κ2N:N'}bis[diiodidozinc(II)] Geometric parameters (Å, º)

**Table:**

I1—Zn1	2.5680 (7)	C11—H11C	0.9800
I2—Zn1	2.5688 (9)	C12—H12A	0.9800
Zn1—N1	2.051 (3)	C12—H12B	0.9800
Zn1—N2^i^	2.058 (3)	C12—H12C	0.9800
Si1—C7	1.859 (4)	C13—C17	1.396 (4)
Si1—C6	1.866 (4)	C13—C14	1.401 (5)
Si1—C8	1.874 (3)	C14—C15	1.386 (4)
Si1—C4	1.889 (3)	C14—H14	0.9500
Si2—C11	1.863 (4)	C15—C16	1.383 (5)
Si2—C12	1.867 (4)	C15—H15	0.9500
Si2—C10	1.877 (4)	C16—H16	0.9500
Si2—C13	1.890 (3)	C17—H17	0.9500
N1—C1	1.342 (4)	C2—C3	1.386 (5)
N1—C5	1.350 (4)	C2—H2	0.9500
N2—C16	1.346 (4)	C3—C4	1.398 (4)
N2—C17	1.353 (4)	C3—H3	0.9500
C1—C2	1.385 (5)	C4—C5	1.395 (4)
C1—H1	0.9500	C5—H5	0.9500
C9—C8	1.539 (5)	C6—H6A	0.9800
C9—C10	1.543 (4)	C6—H6B	0.9800
C9—H9A	0.9900	C6—H6C	0.9800
C9—H9B	0.9900	C7—H7A	0.9800
C10—H10A	0.9900	C7—H7B	0.9800
C10—H10B	0.9900	C7—H7C	0.9800
C11—H11A	0.9800	C8—H8A	0.9900
C11—H11B	0.9800	C8—H8B	0.9900
			
N1—Zn1—N2^i^	101.75 (11)	Si2—C12—H12C	109.5
N1—Zn1—I1	112.18 (9)	H12A—C12—H12C	109.5
N2^i^—Zn1—I1	105.99 (8)	H12B—C12—H12C	109.5
N1—Zn1—I2	103.93 (8)	C17—C13—C14	116.5 (3)
N2^i^—Zn1—I2	110.36 (8)	C17—C13—Si2	120.4 (2)
I1—Zn1—I2	121.03 (2)	C14—C13—Si2	123.1 (2)
C7—Si1—C6	110.83 (18)	C15—C14—C13	120.4 (3)
C7—Si1—C8	109.85 (17)	C15—C14—H14	119.8
C6—Si1—C8	111.23 (15)	C13—C14—H14	119.8
C7—Si1—C4	106.29 (16)	C16—C15—C14	119.0 (3)
C6—Si1—C4	109.39 (16)	C16—C15—H15	120.5
C8—Si1—C4	109.12 (14)	C14—C15—H15	120.5
C11—Si2—C12	109.74 (18)	N2—C16—C15	122.2 (3)
C11—Si2—C10	111.15 (17)	N2—C16—H16	118.9
C12—Si2—C10	110.25 (16)	C15—C16—H16	118.9
C11—Si2—C13	109.42 (16)	N2—C17—C13	123.7 (3)
C12—Si2—C13	107.79 (17)	N2—C17—H17	118.2
C10—Si2—C13	108.42 (14)	C13—C17—H17	118.2
C1—N1—C5	118.2 (3)	C1—C2—C3	118.8 (3)
C1—N1—Zn1	120.1 (2)	C1—C2—H2	120.6
C5—N1—Zn1	121.6 (2)	C3—C2—H2	120.6
C16—N2—C17	118.3 (3)	C2—C3—C4	120.6 (3)
C16—N2—Zn1^i^	120.8 (2)	C2—C3—H3	119.7
C17—N2—Zn1^i^	121.0 (2)	C4—C3—H3	119.7
N1—C1—C2	122.2 (3)	C5—C4—C3	116.1 (3)
N1—C1—H1	118.9	C5—C4—Si1	120.1 (2)
C2—C1—H1	118.9	C3—C4—Si1	123.4 (2)
C8—C9—C10	113.7 (3)	N1—C5—C4	124.1 (3)
C8—C9—H9A	108.8	N1—C5—H5	118.0
C10—C9—H9A	108.8	C4—C5—H5	118.0
C8—C9—H9B	108.8	Si1—C6—H6A	109.5
C10—C9—H9B	108.8	Si1—C6—H6B	109.5
H9A—C9—H9B	107.7	H6A—C6—H6B	109.5
C9—C10—Si2	113.8 (2)	Si1—C6—H6C	109.5
C9—C10—H10A	108.8	H6A—C6—H6C	109.5
Si2—C10—H10A	108.8	H6B—C6—H6C	109.5
C9—C10—H10B	108.8	Si1—C7—H7A	109.5
Si2—C10—H10B	108.8	Si1—C7—H7B	109.5
H10A—C10—H10B	107.7	H7A—C7—H7B	109.5
Si2—C11—H11A	109.5	Si1—C7—H7C	109.5
Si2—C11—H11B	109.5	H7A—C7—H7C	109.5
H11A—C11—H11B	109.5	H7B—C7—H7C	109.5
Si2—C11—H11C	109.5	C9—C8—Si1	115.0 (2)
H11A—C11—H11C	109.5	C9—C8—H8A	108.5
H11B—C11—H11C	109.5	Si1—C8—H8A	108.5
Si2—C12—H12A	109.5	C9—C8—H8B	108.5
Si2—C12—H12B	109.5	Si1—C8—H8B	108.5
H12A—C12—H12B	109.5	H8A—C8—H8B	107.5
			
C5—N1—C1—C2	−0.1 (6)	C14—C13—C17—N2	0.2 (4)
Zn1—N1—C1—C2	177.1 (3)	Si2—C13—C17—N2	−178.8 (2)
C8—C9—C10—Si2	170.5 (2)	N1—C1—C2—C3	0.3 (6)
C11—Si2—C10—C9	−175.7 (2)	C1—C2—C3—C4	−0.6 (6)
C12—Si2—C10—C9	−53.8 (3)	C2—C3—C4—C5	0.7 (5)
C13—Si2—C10—C9	64.0 (2)	C2—C3—C4—Si1	−172.2 (3)
C11—Si2—C13—C17	−96.0 (3)	C7—Si1—C4—C5	−77.1 (3)
C12—Si2—C13—C17	144.7 (2)	C6—Si1—C4—C5	42.6 (3)
C10—Si2—C13—C17	25.4 (3)	C8—Si1—C4—C5	164.5 (3)
C11—Si2—C13—C14	85.1 (3)	C7—Si1—C4—C3	95.4 (3)
C12—Si2—C13—C14	−34.2 (3)	C6—Si1—C4—C3	−144.9 (3)
C10—Si2—C13—C14	−153.5 (2)	C8—Si1—C4—C3	−23.0 (3)
C17—C13—C14—C15	−0.3 (4)	C1—N1—C5—C4	0.2 (5)
Si2—C13—C14—C15	178.6 (2)	Zn1—N1—C5—C4	−176.9 (3)
C13—C14—C15—C16	−0.1 (5)	C3—C4—C5—N1	−0.5 (5)
C17—N2—C16—C15	−1.0 (5)	Si1—C4—C5—N1	172.6 (3)
Zn1^i^—N2—C16—C15	179.8 (2)	C10—C9—C8—Si1	178.6 (2)
C14—C15—C16—N2	0.8 (5)	C7—Si1—C8—C9	173.1 (2)
C16—N2—C17—C13	0.5 (4)	C6—Si1—C8—C9	50.0 (3)
Zn1^i^—N2—C17—C13	179.7 (2)	C4—Si1—C8—C9	−70.7 (3)

Symmetry code: (i) −*x*+1, −*y*+1, −*z*+1.

### Bis{µ-1,3-bis[dimethyl(pyridin-3-yl)silyl]propane-κ2N:N'}bis[diiodidozinc(II)] Hydrogen-bond geometry (Å, º)

**Table:**

*D*—H···*A*	*D*—H	H···*A*	*D*···*A*	*D*—H···*A*
C1—H1···I1^ii^	0.95	3.08	3.805 (4)	135
C15—H15···I2^iii^	0.95	3.27	3.756 (3)	114

Symmetry codes: (ii) *x*+1, *y*, *z*; (iii) −*x*, −*y*+1, −*z*+1.
